# A Rare Case of Orofacial Tuberculosis Mimicking a Buccal Abscess

**DOI:** 10.7759/cureus.98521

**Published:** 2025-12-05

**Authors:** Vidhya Rathnavelu, Soumya Anandan, Rajshri Radhakrishnan, Kalpa Pandya, Ravindran Chinnaswami

**Affiliations:** 1 Oral Pathology and Microbiology, Sri Ramachandra Institute of Higher Education and Research, Chennai, IND; 2 Dentistry, Sri Ramachandra Institute of Higher Education and Research, Chennai, IND; 3 Oral and Maxillofacial Surgery, Sri Ramachandra Institute of Higher Education and Research, Chennai, IND

**Keywords:** anti-tubercular therapy, extrapulmonary tuberculosis, maxilofacial, mycobacterium tuberculosis, orofacial tuberculosis

## Abstract

Tuberculosis (TB) remains a leading cause of infectious disease mortality worldwide, with extrapulmonary manifestations often presenting diagnostic challenges. Orofacial TB, though rare, can mimic other inflammatory or neoplastic conditions due to its non-specific clinical features. We report a case of a 44-year-old female presenting with a persistent, non-tender swelling in the left buccal mucosa. Radiographic imaging suggested a fistulous tract and associated intraosseous changes. Initial antibiotic therapy and extraction of suspect teeth failed to resolve the lesion. Histopathological examination following incisional biopsy revealed granulomatous inflammation with caseating necrosis and Langhans-type giant cells, confirming TB. After ruling out systemic TB, a diagnosis of orofacial TB was made. The patient responded favorably to a six-month anti-tubercular therapy regimen, with complete resolution of symptoms. This case highlights the importance of maintaining a high index of suspicion for TB in persistent orofacial lesions, particularly in endemic areas. A multidisciplinary diagnostic approach is essential for timely and accurate identification to ensure effective treatment outcomes.

## Introduction

Tuberculosis (TB) represents the leading cause of mortality attributable to infectious diseases, with an estimated 1.25 million fatalities recorded in 2023, as reported by the World Health Organization (WHO) [1]. Tuberculosis is an infectious disease caused by the pathogen Mycobacterium tuberculosis, characterized by a chronic granulomatous inflammatory response.

TB manifests in both pulmonary and extrapulmonary sites, including involvement of the orofacial region. Within the orofacial area, any location in the oral cavity and its associated structures, such as the tongue, palate, lips, oral mucosa, jaw bones, sinuses, and the temporomandibular joint (TMJ), may be affected [2]. The clinical presentation of orofacial TB typically includes ulcerative lesions, but may also present as non-ulcerative lesions (patches, plaques) or granulomatous lesions (indurated soft tissue lesions) [3]. Intraoral manifestations often include chronic, persistent ulcers, while the occurrence of swelling is relatively uncommon, even when it is secondary to primary pulmonary TB.

Due to its non-specific clinical symptoms, the diagnosis of orofacial TB poses a significant challenge for healthcare practitioners. A definitive diagnosis can only be achieved through a comprehensive correlation of clinical, radiological, and histopathological assessments. In this context, we present a case report of orofacial TB in a 44-year-old female patient, where the diagnosis was established utilizing a similar diagnostic methodology.

## Case presentation

**Figure 1:**
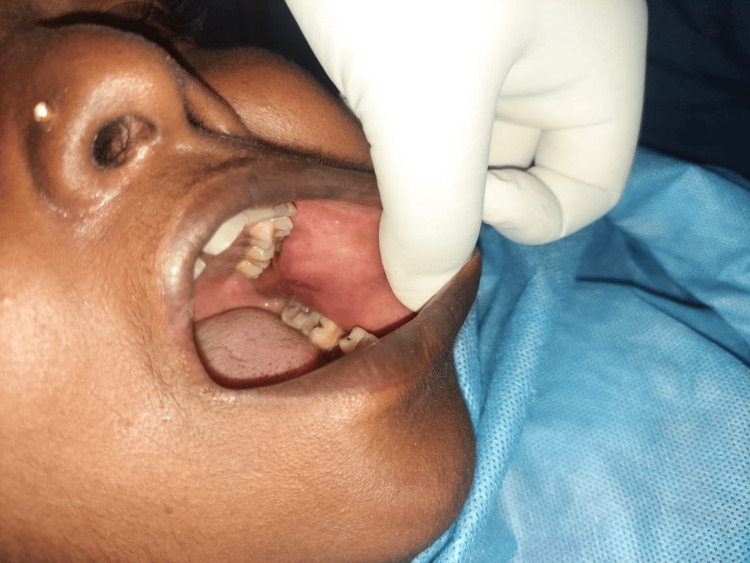
Clinical photograph showing a well-defined, non-tender swelling in the left buccal mucosa adjacent to the 36 region.

A 44-year-old female patient presented with a complaint of swelling localized to the left cheek region persisting for a duration of two months. There was an absence of any history indicative of pain or purulent discharge associated with the swelling. Upon intraoral examination, a solitary swelling measuring 2x3 cm was identified in the left buccal mucosa, situated adjacent to the buccal cusps of teeth 26 and 27 (Figure 1).

**Figure 2:**
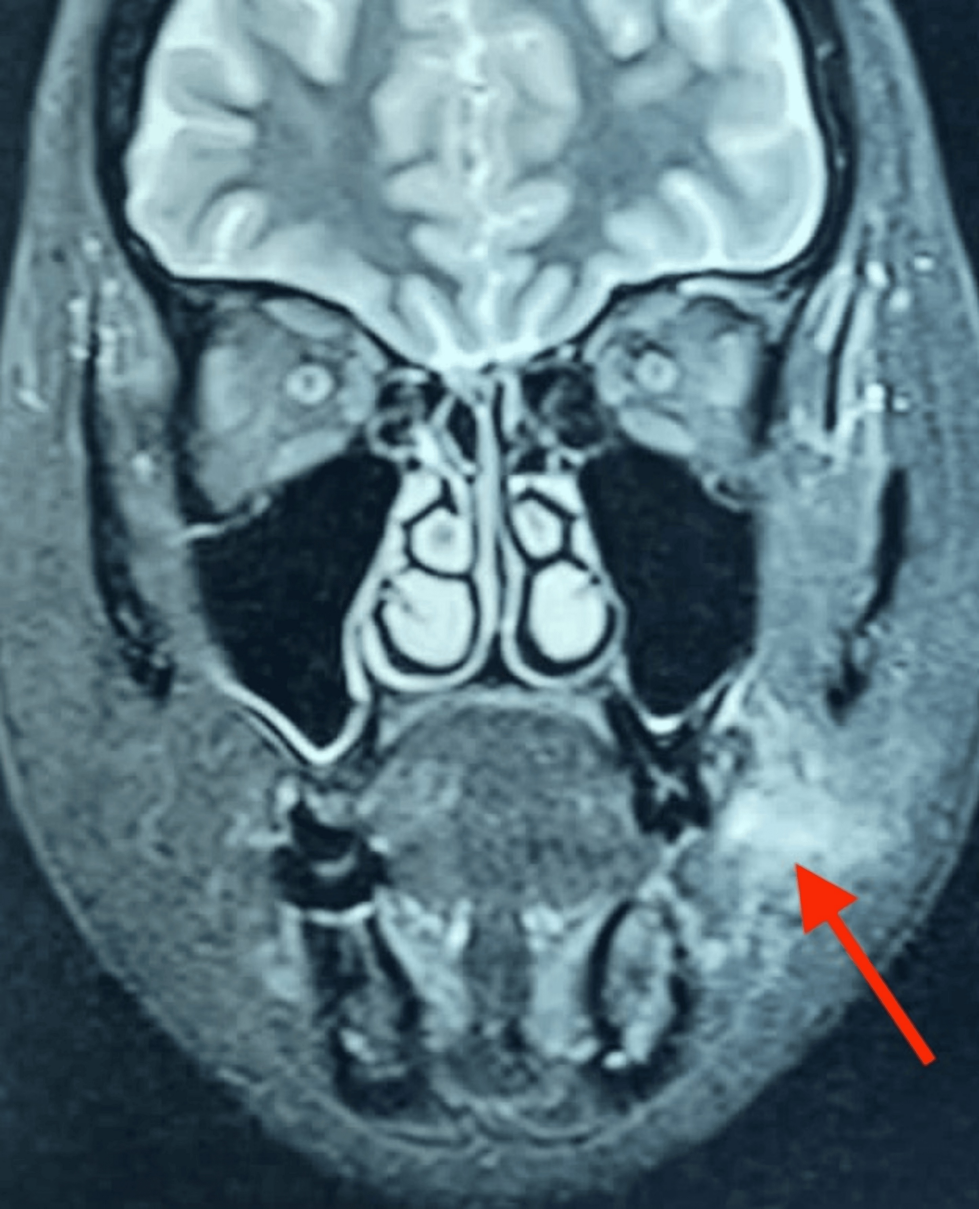
Coronal T2-weighted MRI sequence shows fluid-filled thick sinus on the left side

On palpation, the swelling exhibited a firm consistency, was non-tender, and demonstrated free mobility. Additional findings included dental caries associated with teeth 27 and 28, alongside a clinically absent tooth 36. An intraoral periapical radiograph of tooth 36 disclosed the presence of a retained root stump. Given the submucosal character of the swelling, a Magnetic Resonance Imaging (MRI) scan of the facial region was conducted. T2-weighted MRI sequence elucidated a fluid-filled, thick sinus or fistulous tract in the left cheek area along the subcutaneous plane, superficially extending just beneath the skin and appearing internally to open into the oral cavity near the upper first or second molar tooth. There was also focal intraosseous fluid intensity observed in the alveolar process of the mandible adjacent to the left first molar, likely indicative of specific infective or inflammatory changes (Figure 2).

With a provisional diagnosis of buccal abscess, the extraction of the root stump of tooth 36 and tooth 28 was executed. The fluid was subsequently aspirated from the swelling and sent for cytological examination. The cytological report indicated the presence of pus discharge accompanied by inflammatory cells. The patient was prescribed a regimen combining amoxicillin and clavulanic acid as a post-operative antibiotic protocol.

**Figure 3:**
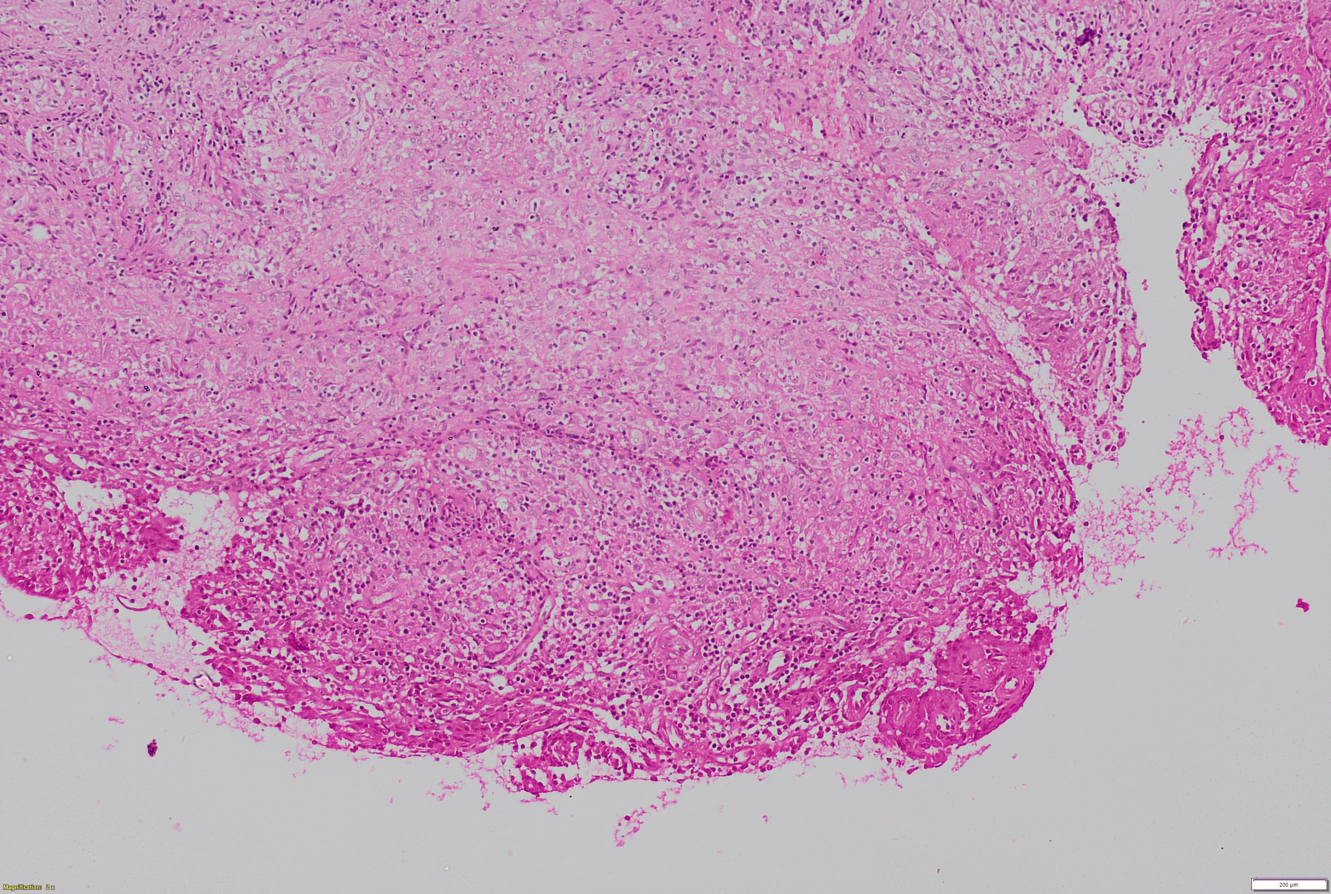
10X view of tuberculosis (TB) showing features of caseating necrosis and a foamy connective tissue stroma.

**Figure 4:**
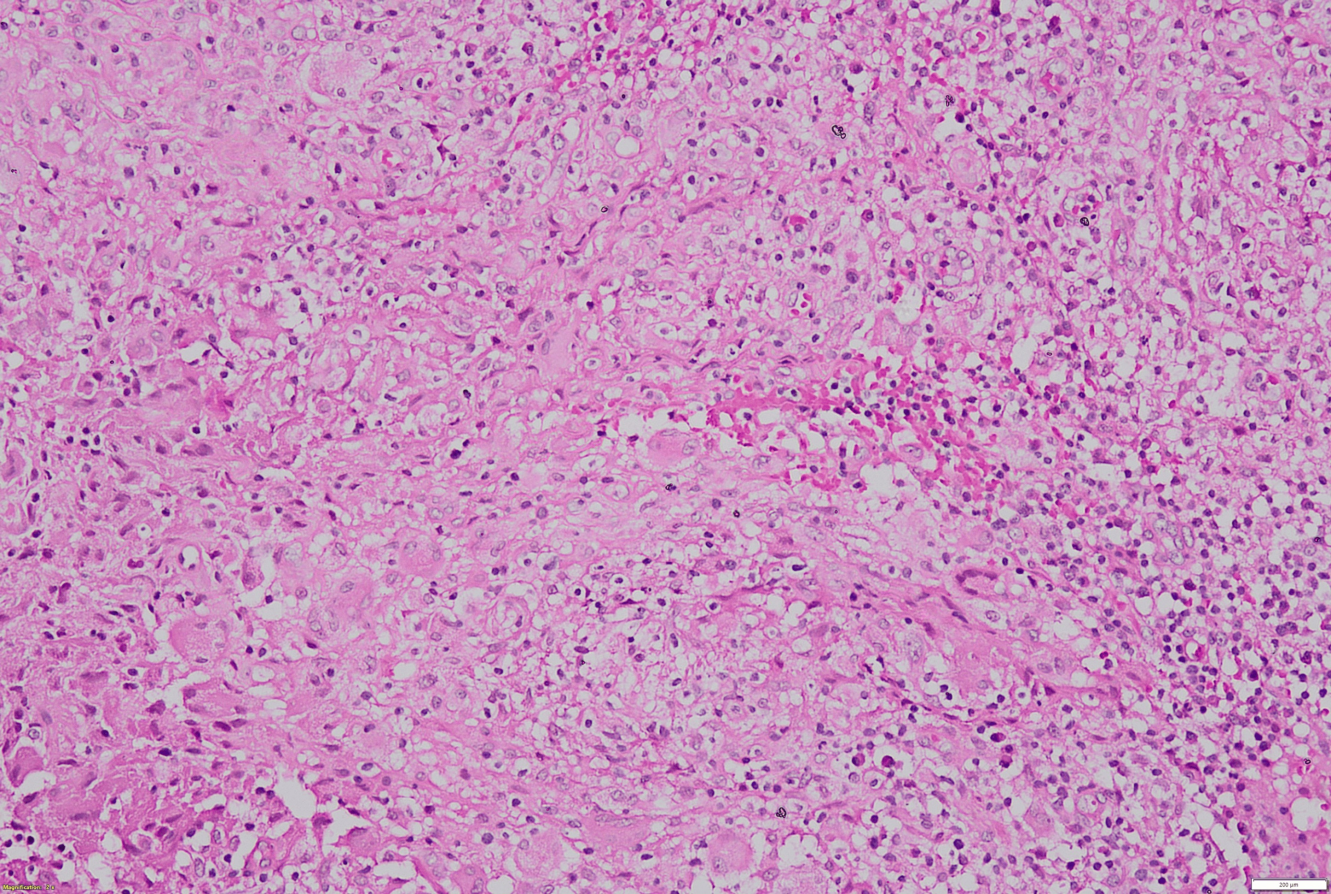
Photomicrograph (H&E stain, ×10) showing granulomatous inflammation with areas of caseating necrosis and foamy connective tissue stroma.

**Figure 5:**
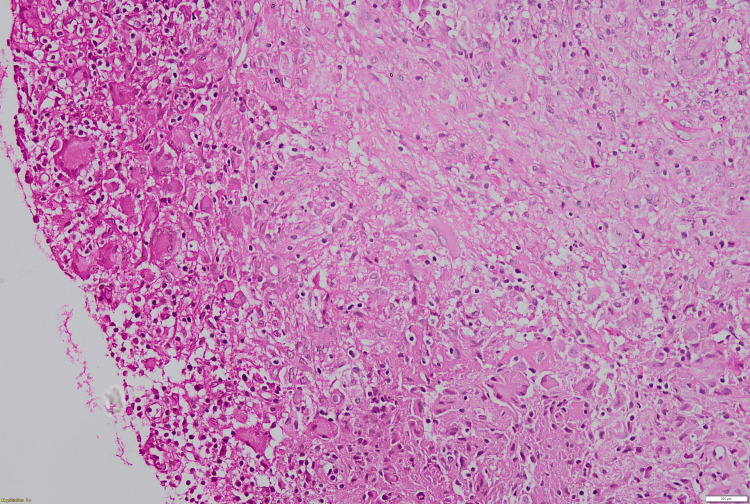
10X view of tuberculosis (TB) showing features of multinucleated giant cells (MGCs)

One week subsequent to the procedure, the absence of any reduction in the swelling necessitated further investigative measures. An incisional biopsy of the swelling was performed. Histopathological evaluation revealed the connective tissue stroma characterized by caseating necrosis and degeneration, accompanied by granulomas, numerous giant cells, and a diverse population of inflammatory cells, including neutrophils, lymphocytes, and extravasated cells (Figures 3, 4, 5).

Consequently, based on the histopathological findings, a diagnosis of orofacial tuberculosis was established. Sputum culture and gene expert were negative for TB. Also, chest X-ray did not reveal any lesions, indicating a history of pulmonary TB. The patient was referred to a physician who initiated a course of an anti-tubercular drug regimen for a duration of six months. Upon a six-month follow-up, the swelling had completely subsided, with a concomitant alleviation of symptoms.

## Discussion

Tuberculosis manifests in both pulmonary and extrapulmonary forms. Among these, oral lesions exhibit a significantly lower prevalence, approximately 0.5 - 1% of individuals afflicted with tuberculosis [4].

Diagnosing orofacial TB presents several challenges due to its rarity and the nonspecific nature of its clinical manifestations, which can easily be mistaken for other conditions such as malignancies or other granulomatous diseases. The difficulty in diagnosis is compounded by the fact that orofacial TB often presents without the classic systemic symptoms of tuberculosis, leading to potential misdiagnosis and delayed treatment. This necessitates a high index of suspicion and a comprehensive diagnostic approach to differentiate it from other orofacial swellings. Orofacial TB can manifest as ulcers, nodules, or diffuse swellings, which are often mistaken for other conditions such as malignancies or chronic infections. These lesions are typically resistant to conventional treatments, which should raise suspicion for TB [5,6].

Histopathological examination remains a cornerstone for diagnosing orofacial TB, with the presence of granulomatous inflammation, epithelioid cells, and Langhan's giant cells being indicative of TB [5,7]. Acid-fast bacilli staining and culture, along with immunological assays, are essential diagnostic tools [7,8].

Once diagnosed, antituberculous therapy (ATT) is effective in resolving orofacial TB lesions. Strict adherence to the treatment regimen is necessary to prevent the development of drug-resistant strains [5,9]. Surgical intervention may be required in cases of unifocal lymph node involvement or when lesions do not respond to medical therapy alone [10].

## Conclusions

Some of the common provisional diagnoses for a firm, non-tender swelling of the buccal wall include space infections, low-grade abscesses, fibromas, lipomas, and minor salivary gland tumors. Granulomatous infections like tuberculosis, although rare, remain a critical differential diagnosis, especially in regions with high TB prevalence or patients with a history of TB exposure.

This case highlights the value of a multidisciplinary diagnostic approach, encompassing clinical evaluation, advanced imaging, and histopathological examination in arriving at an accurate diagnosis. The classic histopathological findings - namely, granuloma with caseating necrosis and Langhans giant cells - were indicative of TB, allowing timely therapeutic intervention with anti-tubercular drug therapy.

This case underscores the importance of considering lateral perspectives during clinical examination. An integrated methodology of clinical, radiological, and laboratory investigations, validated by the histopathological findings, aids in the comprehensive diagnosis of orofacial tuberculosis.

## References

[1] World Health Organization. (2024, October 29) (2024). Global tuberculosis report 2024. https://www.who.int/teams/global-tuberculosis-programme/tb-reports/global-tuberculosis-report-2024.

[2] Bansal R, Jain A, Mittal S (2015). Orofacial tuberculosis: clinical manifestations, diagnosis and management. J Family Med Prim Care.

[3] Jain P, Jain I (2014). Oral manifestations of tuberculosis: step towards early diagnosis. J Clin Diagn Res.

[4] Mahjoub SB (2024). Oral and maxillofacial manifestations of tuberculosis presented to primary health care centres in Port Sudan, Sudan. J Clin Med Surgery.

[5] Dhuvad J, Patel B, Madan S, Dhuvad M (2014). Orofacial tubercular lesions. Indian J Tuberc.

[6] Issa SA, Abdulnabi HA, Jameel ME (2020). Orofacial tuberculosis: a diagnostic challenge. IDCases.

[7] Mignogna MD, Muzio LL, Favia G, Ruoppo E, Sammartino G, Zarrelli C, Bucci E (2000). Oral tuberculosis: a clinical evaluation of 42 cases. Oral Dis.

[8] Favia G, Chiaravalle G, Lacaita MG (1990). [Orofacial tuberculosis: a general and anatomico-clinical analysis of 35 cases]. Minerva Stomatol.

[9] Kishore DN, Geetha NT, Umashankara KV, Rai KK (2014). Submasseteric tuberculous lesion of mandible: report of a case and review of the literature. Case Rep Dent.

[10] Wang H, Wang R, Zheng X (2011). Clinical characteristics of tuberculosis in oral and maxillofacial region. Chin J Prim Med Pharm.

